# Impact of Anatomical Placement on the Accuracy of Wearable Heart Rate Monitors During Rest and Various Exercise Intensities

**DOI:** 10.3390/s26010176

**Published:** 2025-12-26

**Authors:** Masoud Moghaddam, James P. Collins, Caroline E. Gardner, Michael C. Rabel

**Affiliations:** Department of Physical Therapy, School of Pharmacy and Health Professions, University of Maryland Eastern Shore, Hazel Hall, Suite 2082, Princess Anne, MD 21853, USA; jcollins@umes.edu (J.P.C.); cgardner@umes.edu (C.E.G.); mcrabel@umes.edu (M.C.R.)

**Keywords:** accuracy, heart rate monitoring, photoplethysmography, sensor placement, validation study, wearable technology

## Abstract

Purpose: This study evaluated the accuracy of arm-based photoplethysmography (PPG) wearable heart rate (HR) monitors in comparison to a validated chest strap reference across various activity levels. Methods: Twenty-eight healthy adults (14 males, 14 females; aged 23.8 ± 1.1 years) wore six HR monitors: Polar H10 chest strap, Polar Verity Sense on the forearm, Garmin Forerunner on the wrist, and three identical Whoop 4.0 devices placed on the left wrist, forearm, and upper arm. Participants completed rest, warm-up, high-intensity burpees, and graded treadmill exercise. HR data were analyzed using mean absolute percentage error (MAPE), Bland–Altman analysis, concordance correlation coefficient (CCC), and Deming regression. Results: Accuracy was highest at rest and gradually decreased as movement intensity increased. During rest, all devices showed minimal bias and high agreement (CCC > 0.99), with Verity Sense recording the lowest MAPE. In warm-up, Whoop-upper arm and Verity Sense outperformed wrist and Garmin positions, while the Whoop-forearm showed proportional and systematic bias. Burpees resulted in the lowest accuracy across devices (CCC < 0.50), but Whoop-upper arm performed better than other placements. During the Modified Bruce protocol, Verity Sense and Whoop-upper arm had the strongest agreement with the chest strap. Placement of identical Whoop units affected accuracy, with the upper arm outperforming forearm and wrist positions. Conclusions: PPG wearables provided accurate HR monitoring at rest, during warm-up, and during steady-state graded exercise, particularly when positioned proximally (forearm or upper arm). Accuracy declined during short, high-intensity, full-body activities due to motion artifacts. Both the forearm-mounted Verity Sense and the upper-arm Whoop demonstrated the closest agreement with the chest-strap reference. The intra-device comparison of identical Whoop units confirmed that anatomical placement alone significantly affects accuracy.

## 1. Introduction

Heart rate (HR) monitoring provides a non-invasive and accessible means of evaluating cardiovascular health and adaptability to exercise and medical conditions in athletes and clinical patients [1]. For athletes, tracking HR during exercise helps gauge training intensity and stay within optimal target zones, which are often based on a percentage of their maximum heart rate, estimated or obtained through testing [1]. Monitoring resting HR over time also reflects fitness levels, with a lower resting rate indicating a more efficient heart capable of pumping bpd with fewer beats [1,2]. In clinical settings, HR is a key vital sign for assessing cardiovascular health [3]. Abnormal rates, either too high (tachycardia) or too low (bradycardia), may suggest underlying heart issues needing medical attention [1,3]. Additionally, HR monitoring is instrumental in diagnosing and managing conditions such as heart failure, hypertension, arrhythmias, and coronary artery disease [4,5]. A high resting HR independently increases the risk of cardiovascular disease and death, regardless of other factors [6]. Monitoring HR is a foundational component of clinical decision-making [7]. In acute settings, for example, HR guides early mobilization protocols, helps identify signs of physiological instability, and tracks responses to interventions [8,9].

Traditionally, HR monitoring has used electrocardiography (ECG), a well-established and highly accurate method for monitoring HR, whether at rest or during physical activity [10]. It works by detecting the heart’s electrical signals through electrodes placed on the skin, offering valuable insights into the heart’s rhythm, rate, and electrical conductivity [10]. However, ECG systems are widely used in clinical settings and remain the cornerstone of cardiovascular care [10,11]. An HR monitor is a personal device that enables users to track their HR in real-time and record their HR data for future analysis [1]. Modern HR monitors most often record heart signals using either electrical or optical methods [1]. The electrical method utilizes ECG sensors that detect the bioelectric potential generated by electrical activity in the body [12]. In contrast, the optical method utilizes photoplethysmography (PPG) sensors, which employ light-based technology to detect changes in blood volume within the skin’s microvasculature [13,14].

Electrical HR monitors generally consist of two primary components: a monitor and a receiver [1]. The monitor is typically secured using chest straps positioned to detect the electrical activity of the heart. These straps contain electrodes that must maintain contact with the skin to capture electrical signals accurately [1,15]. Upon detecting a heartbeat, the monitor detects the electrical signal, and an integrated transmitter within the strap sends out a radio signal. The receiver, which may be a smartwatch, a smartphone, or a compatible alternative device, captures the transmitted radio signal. Subsequently, the receiver processes this signal to compute and display HR in real-time [1,16]. The Polar H10 (Polar Electro Oy, Kempele, Finland) is a widely used chest strap HR monitor known for its proven accuracy and reliability in recording HR and monitoring HR variability during rest and exercise [16]. Multiple studies have indicated that the Polar H10 demonstrates high accuracy and reliability in measuring HR and linear HR variability parameters compared to ECG during rest and different activities [1,16,17]. It can serve as a useful tool for monitoring these metrics in practical settings, including clinical and sports applications [1,16,17]. However, it is limited in analyzing non-linear HR variability and cannot produce detailed ECG waveform analyses [16].

A standard photoplethysmography (PPG) HR monitor comprises a light source and a photodetector. The light source emits light directed toward the tissue, while the photodetector detects the reflected light, which is indicative of changes in blood flow [13,18]. Similar to ECGs, PPG waves can help diagnose cardiac arrhythmias or irregular heartbeats, as they reliably monitor cardiac and respiratory activities [13]. Most PPG sensors mainly use either an infrared light-emitting diode (IR-LED) or a green LED as their primary light source. IR-LEDs generally target blood flow in deeper tissues, such as muscles, while green LEDs focus on measuring oxygen absorption in oxyhemoglobin (oxygen-rich blood) and deoxyhemoglobin (oxygen-poor blood) [13,19]. Green LEDs are frequently utilized in HR monitors equipped with PPG sensors due to their superior tissue penetration and enhanced accuracy in readings by minimizing motion artifacts [14]. Most PPG sensors also include a photodetector to measure the intensity of reflected light, which is used to calculate changes in blood volume. Previous studies have shown that factors such as sensor placement, contact pressure, mechanical movement artifacts, and body posture affect PPG recordings [14].

**Figure 1:**
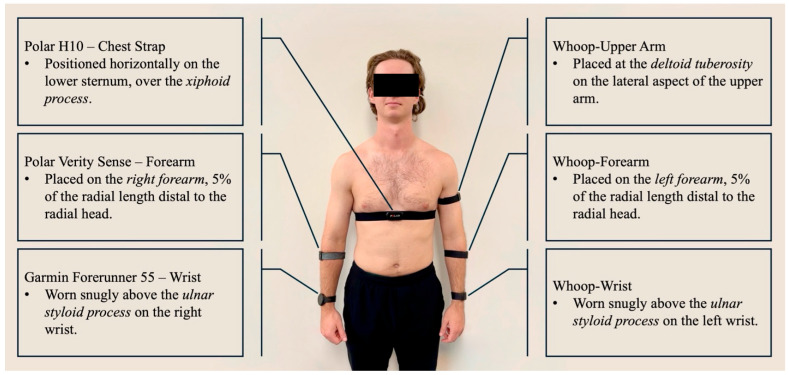
Device placement for all six HR monitors. The reference chest strap (Polar H10) was positioned at the lower sternum, while PPG-based wearables were placed on the wrist, forearm, and upper arm according to manufacturer recommendations.

The placement of the LED and photodetector is a critical factor that affects signal quality and robustness against motion artifacts. Researchers are exploring various sites to optimize the placement of PPG sensor wearables, including the finger [20,21], wrist [22], brachium [14], earlobe [23], and external ear cartilage [24]. Studies suggest that applying moderate pressure between the sensor and the skin improves PPG signal quality. Insufficient pressure results in poor contact and weak signal quality, whereas excessive pressure can disrupt the signal by occluding blood vessels [25,26,27,28]. Despite these investigations, a lack of consensus persists regarding the optimal anatomical placement in dynamic or clinically constrained environments [14]. A key feature of this study is an intra-device, multi-site comparison involving three identical wristband sensors worn simultaneously on the same arm (wrist, forearm, upper arm, see Figure 1). This eliminates differences caused by placement, hardware, or firmware and allows for conclusions about how site effects generalize, regardless of brand or model. These results can guide the design of multi-site wearables, including upper-arm and forearm bands for applications that need higher accuracy during movement. Given the growing use of wearables with PPG technology and the need for reliable alternatives to chest strap monitors, this study aimed to assess the agreement and accuracy of arm-based wearable devices placed at the wrist, forearm, and upper arm compared to a validated chest strap monitor (Polar H10) during varied activity intensities.

We hypothesized that all arm-based wearables would demonstrate acceptable agreement with the chest strap (Polar H10), with those on the wrist offering the highest agreement. This assumption was based on the design goal of most consumer-grade devices, which are optimized for wrist use due to accessibility and user comfort. The wrist is the most common location for consumer HR wearables because of ease of use and consistent positioning for daily wear [3,14]. However, subsequent evidence—including our results—suggests that wrist sites are also more prone to motion artifacts and variations in strap tension, which can reduce PPG signal quality and accuracy [15,21].

## 2. Methodology

### 2.1. Experimental Design

This study employed a within-subjects design to assess the accuracy of multiple arm-based wearable HR monitors compared to a validated chest strap monitor (Polar H10, Polar Electro Oy, Kempele, Finland) across different levels of physical activity. After obtaining informed consent, participants completed a health screening that included resting blood pressure and the Physical Activity Readiness Questionnaire (PAR-Q). Demographic and anthropometric data, such as height, weight, age, and body composition (InBody 270, InBody Co., Ltd., Seoul, Republic of Korea), were recorded. Participants were then familiarized with the testing procedures and protocol.

Each participant was fitted with six wearable devices: three identical Whoop 4.0 bands (Whoop Inc., Boston, MA, USA) placed on the left wrist (proximal to the ulnar styloid process), left forearm (5% of radial length distal to the radial head), and left upper arm (over the deltoid tuberosity); one Polar Verity Sense (Polar Electro Oy, Kempele, Finland) on the right forearm (5% of radial length distal to the radial head); one Garmin Forerunner 55 (Garmin Ltd., Olathe, KS, USA) on the right wrist (proximal to the ulnar styloid process); and one Polar H10 chest strap placed over the xiphoid process (see Figure 1). The same researcher applied all sensors to ensure consistent placement and contact pressure, and each device was checked for proper fit and signal quality before testing.

**Figure 2:**
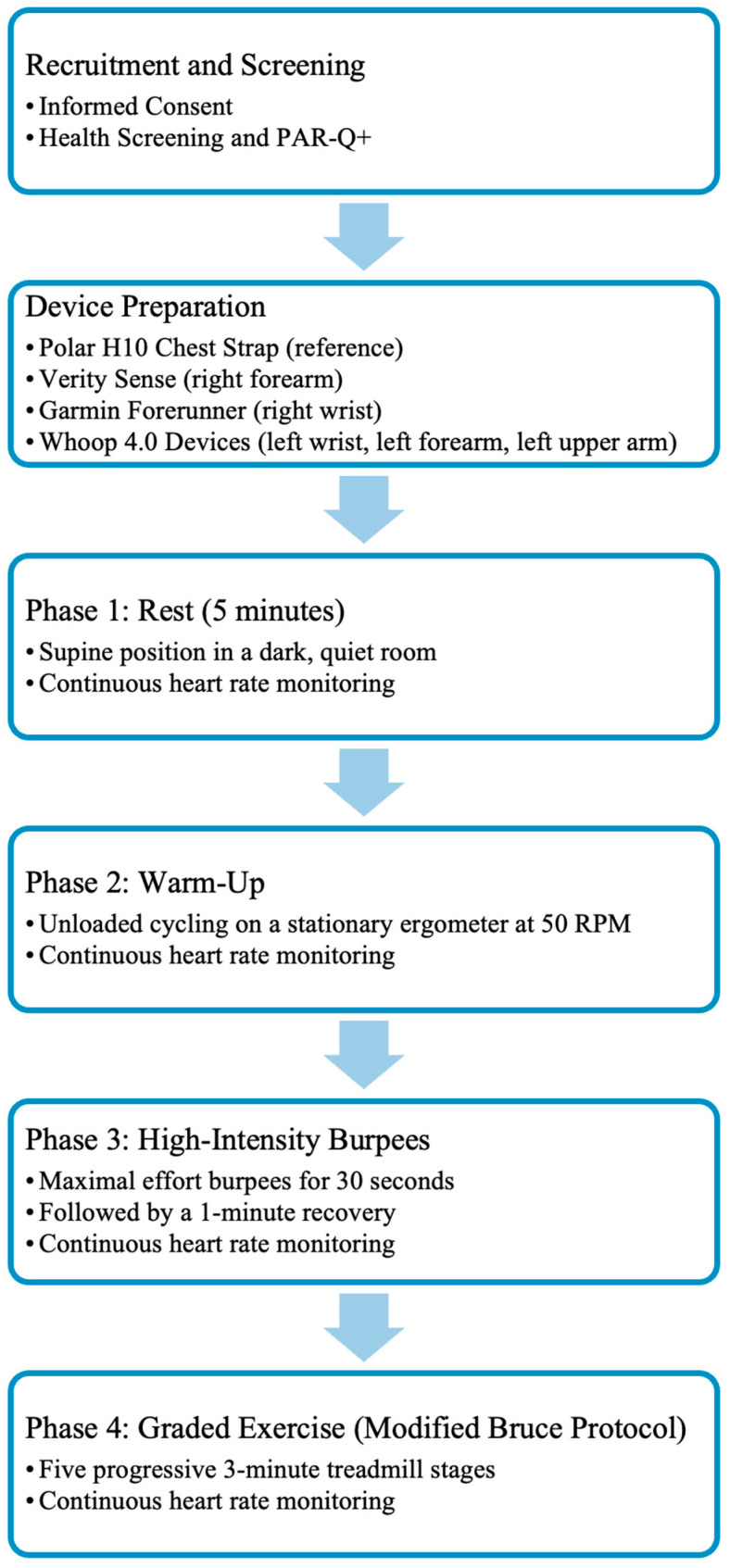
Overview of the experimental workflow from participant recruitment to the graded exercise phase. After informed consent and health screening, participants were fitted with a chest strap reference device and five PPG-based wearables. They then completed four consecutive activity phases: rest, warm-up, high-intensity burpees, and graded exercise. Heart rate was recorded continuously during each activity.

Participants completed four consecutive activity phases while HR was continuously recorded: five minutes of supine rest in a quiet, dark room; a five-minute stationary cycling warm-up at 50 revolutions per minute (RPM); a 30-s bout of maximal-effort burpees; and the first five stages of the Modified Bruce treadmill protocol (see Figure 2). All HR data were captured using the VO2 Master Manager application (VO2 Master Inc., Vernon, BC, Canada) via Bluetooth. Transitions between each activity phase were marked in real time using the application’s event-flagging feature to ensure accurate synchronization for subsequent analysis.

During rest, participants remained supine and motionless in a dark, quiet environment to minimize motion artifacts and autonomic variability. The warm-up involved unloaded cycling at 50 RPM, with seat height adjusted individually to ensure a comfortable position. Immediately afterward, participants performed a 30-s maximum-effort bout of burpees followed by a one-minute recovery, designed to mimic short, full-body, high-intensity exercise. After a five-minute rest, participants completed five progressive stages of the Modified Bruce treadmill protocol, which gradually increased speed and incline to produce graded cardiovascular responses. All start and stop points were precisely time-stamped within the recording software.

### 2.2. Participants

Twenty-eight healthy adults (23.75 ± 1.11 years; 14 males, 14 females) participated in the study (see Table 1). These participants had consistently engaged in an exercise program for at least 30 min, three times per week, over the past six months. Before enrollment, all participants underwent a thorough health screening process to ensure they were in good health, free from any signs or symptoms of disease, and without any musculoskeletal injuries within the previous six months. Participants were instructed to abstain from exercise-related activities, caffeine, and alcohol for 24 h before the testing session. Each participant signed an informed consent document in accordance with the Declaration of Helsinki, acknowledging potential risks and harm. The study was approved by the Institutional Review Board of the University of Maryland Eastern Shore (protocol #04-2025-001).

### 2.3. Statistical Analysis

The normality of HR and HR error data was assessed using the Shapiro–Wilk test. Due to violations of normality, non-parametric tests were used for inferential comparisons. To calculate HR error across different devices and conditions, we selected mean absolute percentage error (MAPE) because it is scale-free and allows comparison across rest-exercise HR ranges. To analyze differences in HR error (MAPE) across the five wearables (Whoop wrist, Whoop forearm, Whoop upper arm, Polar Verity Sense, and Garmin Forerunner 55) during each activity condition (rest, warm-up, burpees, and graded treadmill exercise), Friedman’s test for repeated measures was performed. When significant differences were found, follow-up pairwise comparisons were conducted using Wilcoxon signed-rank tests with Bonferroni corrections to adjust for multiple comparisons.

Assessment of agreement between each device and the chest strap (Polar H10) was conducted using three complementary methods: Bland–Altman analysis, Concordance Correlation Coefficient (CCC), and Deming regression. Furthermore, MAPE was calculated as a descriptive error metric for each device and condition. In the Bland–Altman analysis, systematic (fixed) bias was identified when the mean difference between methods deviated significantly from zero, while proportional bias was assessed by regressing the difference against the mean HR and was considered present when this slope was statistically significant [29]. For Deming regression, 95% confidence intervals (CIs) for the slope and intercept were calculated as the estimated coefficient ± 1.96 × standard error (SE), aligning with prior statistical recommendations [29,30]. In the Deming regression analysis, systematic bias was defined as a statistically significant intercept different from 0, indicating a consistent over- or underestimation across the measurement range, and proportional bias was defined as a slope significantly different from 1.0, indicating that the magnitude of error changed with HR intensity (e.g., greater overestimation at higher HRs or underestimation at lower HRs) [30].

All non-parametric analyses and Bland–Altman plots were performed using IBM SPSS Statistics (Version 28.0, IBM Corp., Armonk, NY, USA), while CCC and Deming regression analyses were conducted using Python (version 3.11). The alpha level (α) for statistical significance was set at 0.05, and all results were reported as mean ± standard deviation (SD) or slope estimates with corresponding *p*-values.

## 3. Results

### 3.1. During 5-Minute Rest

**Figure 3:**
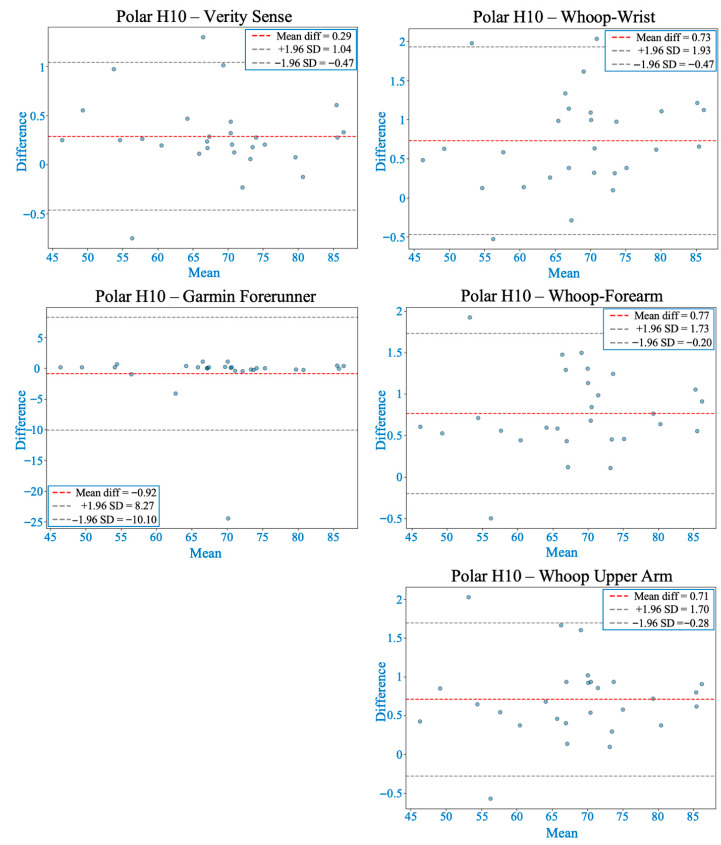
Bland–Altman plots comparing heart rate from each wearable device with the Polar H10 during rest. The red dashed line shows the mean bias, and gray dashed lines represent the 95% limits of agreement (LOA). All devices demonstrated acceptable agreement, with smaller biases and narrower LOA generally observed at proximal arm placements compared to the wrist.

**Figure 4:**
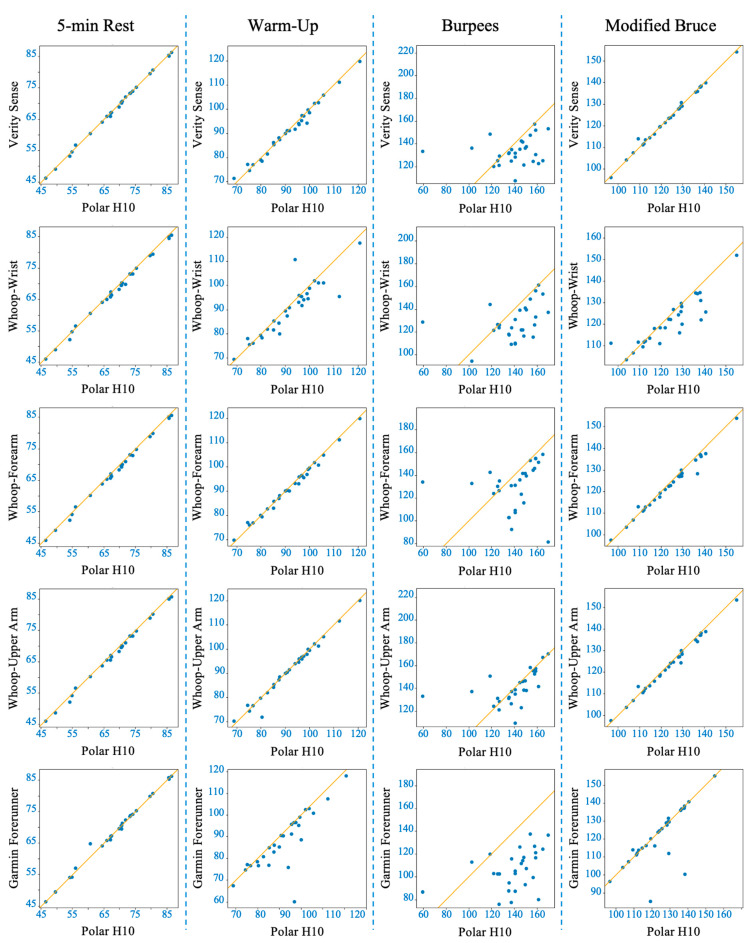
Deming regression plots comparing heart rate (HR) measurements from each wearable device to the Polar H10 reference across four activity conditions (columns: 5-min rest, 5-min warm-up, 30-s burpees + 1-min recovery, Modified Bruce graded exercise test). Rows represent individual devices (Polar Verity Sense, Whoop-wrist, Whoop-forearm, Whoop-upper arm, Garmin Forerunner 55). The solid line indicates the line of best fit from the Deming regression.

Shapiro–Wilk tests confirmed non-normal MAPE distributions across devices (*p* < 0.05); therefore, non-parametric analyses were applied. The Friedman test revealed significant inter-device differences (χ^2^(4) = 62.2, *p* < 0.001). Post hoc comparisons indicated that the Verity Sense (forearm optical armband) produced the lowest error (MAPE ≈ 1.6 ± 0.8%) and the strongest agreement with the Polar H10 chest-strap reference, followed by the Whoop-upper arm and Whoop-forearm. Whoop-wrist and the Garmin Forerunner 55 exhibited the highest error values (see Table 2). Bland–Altman analysis demonstrated minimal bias across all devices (mean bias < 1 bpm, 95% limits of agreement [LOA] within ±10 bpm) (see Figure 3 and Table 3). Concordance correlation coefficients confirmed near-perfect agreement for all placements (ρc ≥ 0.99), with the highest concordance observed for the Verity Sense and the Whoop upper-arm placement (see Table 4). Deming regression slopes approximated 1.0 with non-significant intercepts (*p* > 0.05), indicating strong proportional agreement and no systematic bias (see Figure 4 and Table 5). Collectively, these findings show that all wearables provided accurate HR estimation at rest, but proximal placements—particularly the Verity Sense and Whoop-upper arm—yielded the most precise and stable readings, whereas wrist sites were slightly more variable.

### 3.2. During 5-Minute Warm-Up

**Figure 5:**
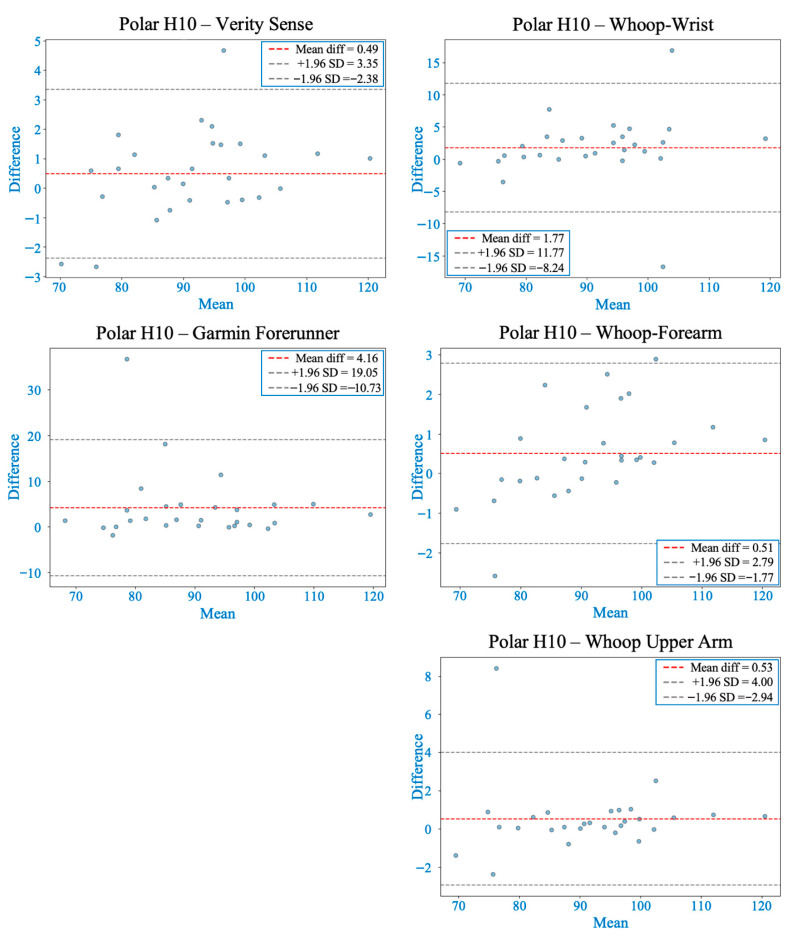
Bland–Altman plots comparing heart rate from each wearable device with the Polar H10 during warm-up. The red dashed line indicates the mean bias, and gray dashed lines represent the 95% limits of agreement (LOA). Most devices showed acceptable agreement, although variability increased compared to rest. Verity Sense and the Whoop-upper arm showed stable agreement with relatively narrow LOA, while Whoop-forearm exhibited proportional and systematic bias. The wrist and Garmin placements displayed wider LOA and greater error variability.

The Friedman test showed significant differences in MAPE between devices (χ^2^(4) = 42.07, *p* < 0.001). Post hoc analyses indicated that the Whoop-upper arm exhibited the lowest error and strongest overall agreement with the chest-strap reference, followed closely by the Verity Sense and Whoop-forearm. In contrast, Whoop-wrist and Garmin displayed greater error variability (see Table 2). Bland–Altman analyses showed that Verity Sense and Whoop-upper arm had narrow limits of agreement (≈±4 bpm) and minimal bias (<1 bpm), while Whoop-forearm demonstrated proportional and systematic bias, suggesting increased overestimation with rising HR. Wrist-based monitors, particularly Garmin, exhibited broader limits (±10–19 bpm), reflecting less precision (see Figure 5 and Table 3). Concordance correlation coefficients were high for Verity Sense (ρc = 0.9912) and both proximal Whoop placements (ρc = 0.9940–0.9879) but lower for wrist-based devices (ρc ≤ 0.89) (see Table 4). Deming regression slopes approximated 1.0 for Verity Sense and Whoop-upper arm, while Whoop-forearm and Garmin showed proportional bias and slight fixed offsets (see Figure 4 and Table 5). Collectively, these results indicate that during light-to-moderate cycling, PPG wearables placed on the upper arm or forearm yield the most accurate and stable HR estimates, whereas wrist sites remain more susceptible to motion artifacts and variable contact pressure.

### 3.3. During 30-Second Burpees with 1-Minute Recovery

**Figure 6:**
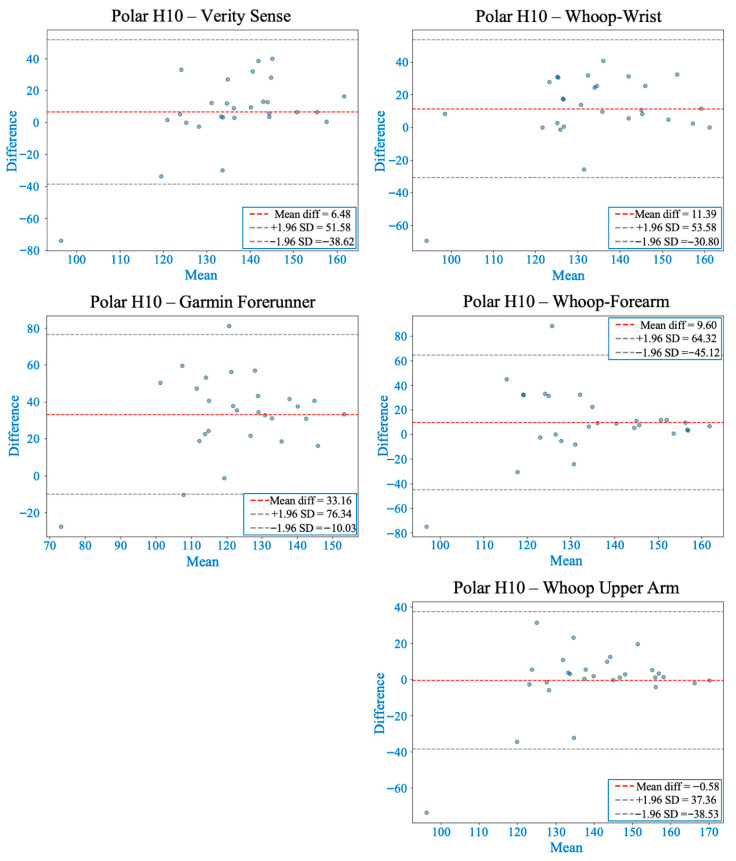
Bland–Altman plots comparing heart rate from each wearable device with the Polar H10 during 30 s of burpees followed by 1 min of recovery. The red dashed line marks the mean bias, while gray dashed lines show the 95% limits of agreement (LOA). Whoop-upper arm showed the smallest bias but displayed proportional and systematic bias at higher heart rates. Verity Sense also showed proportional and systematic bias, whereas Whoop-wrist and Whoop-forearm had larger mean differences without significant regression bias. Garmin had the highest mean bias and the widest LOA, indicating less accuracy during high-intensity, ballistic movements.

The Friedman test indicated significant inter-device differences in MAPE (χ^2^(4) = 37.23, *p* < 0.001). Post hoc comparisons showed that the Whoop-upper arm yielded the lowest error and best agreement with the chest-strap reference, followed by Verity Sense and the other Whoop placements, whereas the Garmin wrist device exhibited the highest error (see Table 2). Bland–Altman analysis revealed markedly wider limits of agreement across all devices compared with rest or warm-up, reflecting the greater motion artifacts of explosive movements. Verity Sense and Whoop-upper arm demonstrated small mean bias (<1 bpm) but showed proportional and systematic bias, indicating overestimation at higher HR values. Wrist and forearm placements showed moderate bias and broader 95% limits (≈±40–60 bpm), while Garmin presented the largest variability (bias ≈ 33 bpm) (see Figure 6 and Table 3).

Concordance correlation coefficients were notably reduced under high-intensity conditions, with Whoop-upper arm achieving the highest agreement (ρc = 0.46) and all other devices below 0.35, highlighting the sensitivity of PPG signals to movement interference (see Table 4). Deming regression confirmed proportional and fixed biases for most devices, particularly the wrist and forearm placements, while Garmin showed proportional bias without a fixed offset (see Figure 4 and Table 5). Collectively, these results indicate that brief, full-body dynamic activity substantially reduces PPG accuracy across all wearables; however, proximal upper-arm placement remains relatively more reliable than wrist or forearm sites.

### 3.4. During Modified Bruce Graded Exercise Testing

**Figure 7:**
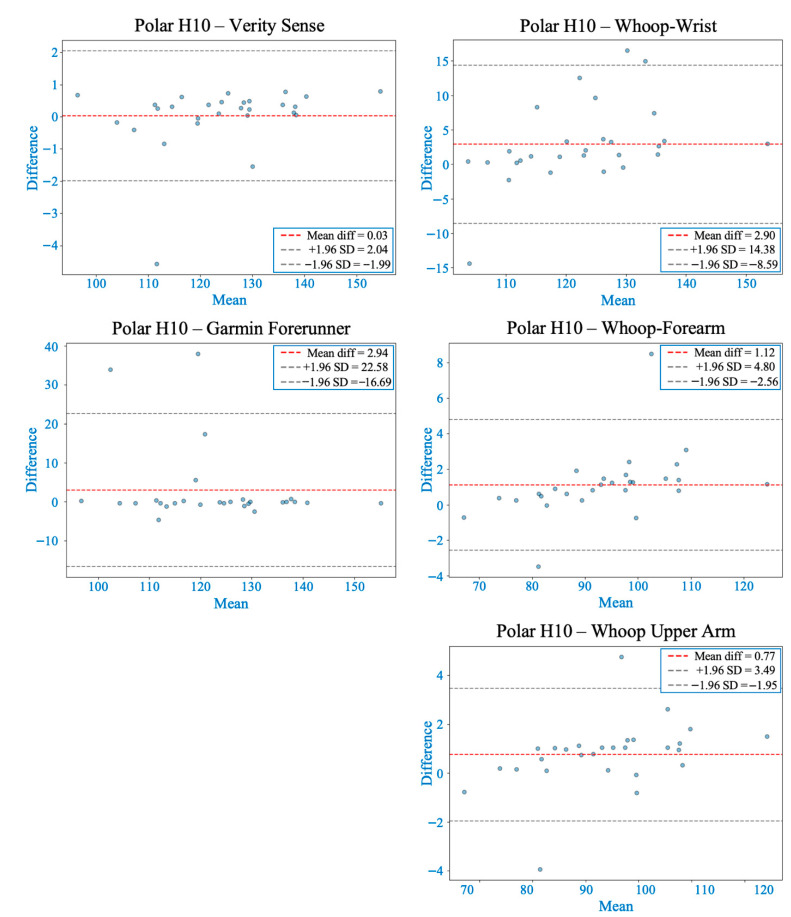
Bland–Altman plots comparing heart rate measurements from each wearable device with the Polar H10 during the Modified Bruce graded exercise protocol. The red dashed line indicates the mean bias, and gray dashed lines represent the 95% limits of agreement (LOA). Verity Sense showed the closest agreement with minimal bias and narrow LOA, followed by the Whoop-upper arm with slight proportional bias. Whoop-forearm and Whoop-wrist demonstrated both proportional and systematic bias, while Garmin exhibited the widest LOA, indicating higher variability across intensities.

The Friedman test showed significant differences in MAPE among devices (χ^2^(4) = 41.09, *p* < 0.001). Post hoc analyses revealed that the Verity Sense and Whoop-upper arm had the lowest error and the strongest agreement with the chest-strap reference, followed by Whoop-forearm. In contrast, Whoop-wrist and Garmin devices exhibited more variability (see Table 2). Bland–Altman analyses indicated that Verity Sense had the smallest bias (<0.1 bpm) and the narrowest limits of agreement (±2 bpm), while Whoop-upper arm also performed well (bias ≈ 0.8 bpm, LOA ± 3.5 bpm) with minor proportional bias at higher intensities. Forearm and wrist placements showed progressively larger bias and wider limits (up to ±14 bpm), reflecting the effects of motion artifacts and tissue deformation during treadmill walking. Garmin displayed the broadest limits (±20 bpm), indicating substantial variability despite lacking a fixed bias (see Figure 7 and Table 3).

Concordance correlation coefficients confirmed high agreement for Verity Sense (ρc = 0.997) and Whoop-upper arm (ρc = 0.992), moderate for Whoop-forearm (ρc = 0.985), and notably lower for wrist-worn devices (ρc ≤ 0.85) (see Table 4). Deming regression results supported these trends: slopes near 1.0 for Verity Sense and Whoop-upper arm reflected proportional accuracy, while Whoop-forearm and wrist sites showed both fixed and proportional bias, and Garmin consistently overestimated HR across workloads (see Figure 4 and Table 5). Collectively, these findings show that during graded treadmill exercise, proximal arm sites—particularly the forearm and upper arm—provide stable and accurate PPG signals, whereas wrist-worn devices are more prone to motion-related error as exercise intensity increases.

## 4. Discussion

This study investigated the accuracy of PPG-based wearable HR monitors placed at different arm sites and compared their performance against a validated chest strap across various activity intensities. Accuracy was highest during resting and low-movement conditions and gradually declined as exercise intensity and movement amplitude increased, due to motion artifacts, changes in tissue compression, and fluctuations in optical coupling between the sensor and skin [21,31]. Throughout all activities, proximal arm placements (upper arm and forearm) consistently showed better agreement with the chest-strap reference than distal wrist sites, confirming that anatomical location significantly affects optical signal stability [13,32]. These findings likely result from physiological and mechanical differences among regions—proximal sites have more soft-tissue padding, deeper vasculature, and less motion relative to the sensor. In contrast, the wrist is dominated by tendons, bony prominences, and frequent joint movement, which can disrupt signal consistency [14,19]. These trends are summarized across activities in Table 2, Table 3, Table 4 and Table 5 and Figure 3, Figure 4, Figure 5, Figure 6 and Figure 7.

In addition to anatomical placement, device-specific optical and algorithmic factors also shaped measurement accuracy. Devices incorporating multi-sensor arrays, higher sampling frequencies, and motion-compensation algorithms—such as the Polar Verity Sense and Whoop 4.0—exhibited stronger correspondence with the chest strap, particularly when worn proximally [14,21,33]. Conversely, devices with simpler single-wavelength configurations and slower signal averaging, such as the Garmin Forerunner 55, showed greater variability during dynamic activity [14,20]. Collectively, these findings highlight that PPG performance depends on a combination of biophysical context (tissue structure and movement) and device design (sensor configuration, optical power, and filtering capacity), underscoring the need to align wearable design and placement strategy with the physiological conditions of intended use [14,31,32].

### 4.1. Accuracy Trends Across Activity Intensities

The results showed a clear inverse relationship between exercise intensity and PPG accuracy. During rest and minimal movement, all wearables closely tracked HR, showing minimal bias and almost perfect agreement with the chest strap (ρc ≥ 0.99). As activity intensity increased, accuracy gradually declined, reflecting the combined effects of motion-induced artifacts, tissue deformation, and temporary loss of optical coupling between the sensor and skin [14,31]. The decrease in accuracy was most significant during short, high-acceleration movements, such as burpees, where rapid changes in limb velocity and contact pressure disrupt the pulsatile waveform and reduce the signal-to-noise ratio [21,34]. Conversely, accuracy partially recovered during the graded treadmill protocol, despite higher absolute HR levels. This improvement likely results from the rhythmic and repetitive nature of walking gait, which enables temporal filtering algorithms to isolate the cardiac signal from motion noise better [31,32]. These findings emphasize that PPG accuracy depends not only on exercise intensity but also on movement type and mechanical consistency. Dynamic, multidirectional movements—characterized by sudden acceleration, joint rotation, and intermittent skin stretch—produce unstable optical conditions that degrade the PPG waveform through variable light scattering and inconsistent sensor-skin contact [14,31]. In contrast, cyclic motions involving steady muscle tension and consistent pressure, such as walking or cycling, promote more stable optical coupling and improved signal fidelity [20,32]. Although proximal placements (upper arm and forearm) remained more reliable than wrist sites across all conditions, they experienced reduced accuracy during ballistic, full-body actions due to motion artifacts and transient changes in tissue perfusion inherent in vigorous activity [21,34].

### 4.2. Physiological and Optical Mechanisms of Placement Differences

The higher accuracy of proximal placements likely results from both anatomical and optical factors. The upper arm and forearm have thicker soft-tissue layers and larger, deeper arterial vessels (e.g., the brachial and radial arteries), which produce a stronger pulsatile blood volume signal and greater optical absorption depth than the wrist [31,32,34,35]. These tissues also demonstrate more stable mechanical behavior, with less displacement and deformation during movement, enabling steadier sensor-skin contact and reducing motion-related optical noise [14,21]. Conversely, the wrist has thinner skin, less subcutaneous fat, and more tendinous and bony structures (the radius and ulna), which alter the local refractive index and scatter light, causing irregular photon trajectories during flexion and extension [13,31,36]. Hemodynamically, proximal regions also experience more stable perfusion and fewer vasoconstrictive fluctuations, which support consistent arterial pulse amplitude and better signal-to-noise ratios, especially under low-motion or steady-state conditions [31,35,37]. Additionally, optical scattering depends on tissue composition and wavelength penetration: muscle-rich areas such as the upper arm allow deeper light transmission and less specular reflection, while thinner, tendon-dominant regions such as the wrist mainly reflect superficial light that is more affected by ambient interference and movement [14,34,36,37]. Therefore, these anatomical and optical features explain why devices placed on the upper arm or forearm show narrower limits of agreement and lower error compared to distal placements.

### 4.3. Inter-Device Differences: Optical Design and Signal Processing

Aside from anatomical placement, differences in optical design and signal processing greatly influenced device accuracy. The Polar Verity Sense and Whoop 4.0 feature more advanced optical and algorithmic systems than the Garmin Forerunner 55, which uses a simpler, consumer-grade green LED array designed primarily for general wellness tracking rather than high-precision physiological monitoring. The Verity Sense uses dual-wavelength emitters (green and red LEDs) and a multi-element photodiode array, allowing for deeper tissue penetration and better compensation for differences in skin pigmentation and tissue thickness [14,31,38]. Dual-wavelength sensing enhances optical depth profiling and helps the device distinguish arterial pulsation from venous and motion-related noise [38,39,40]. It also integrates adaptive filtering and motion-compensation algorithms that adjust signal weighting during movement, improving temporal stability and reducing motion-induced baseline drift [38,39,40]. Similarly, the Whoop 4.0 combines multi-LED green and infrared sensors with accelerometer-synchronized motion correction, enabling better signal reconstruction during rhythmic activity [38,39,40]. When placed close to the body, this multi-sensor setup achieved accuracy comparable to the Verity Sense. However, performance dropped at the wrist, indicating that even advanced algorithms cannot fully compensate for anatomical instability or contact differences at distal sites [21,31,36]. In contrast, the Garmin Forerunner 55 uses a single-wavelength green LED system with a lower sampling frequency and less complex motion filtering. Its algorithms prioritize power efficiency and HR averaging, resulting in delayed HR tracking and larger deviations during rapid HR changes, especially during dynamic or high-intensity activities [13]. These technical differences explain its wider limits of agreement and lower concordance coefficients compared to the other devices. Nevertheless, these findings demonstrate that both placement stability and sensor design determine real-world PPG accuracy. See Table 3, Table 4 and Table 5 and Figure 4 for agreement and regression summaries across activities.

### 4.4. Synthesis Across Activity Intensities

Across different activity conditions, the measurement deviation consistently followed this order: rest, warm-up, graded exercise, and high-intensity burpees. This pattern reflects the combined influence of motion artifacts, tissue deformation, and variations in optical coupling on the stability of the PPG signal. At rest, all devices showed strong proportional agreement and minimal bias, indicating that even consumer-grade wearables can achieve near-reference accuracy in static or low-motion conditions [14,32]. During light and moderate activity, proximal placements such as the Verity Sense and Whoop upper-arm configurations maintained higher accuracy because of better sensor-skin contact and the detection of deeper vascular pulsations [21,31]. Conversely, distal sites began to show a proportional bias as rhythmic movements induced microdisplacements and variations in contact pressure, altering the optical path length and scattering geometry [13,20]. Under high-intensity, ballistic conditions such as burpees, both motion amplitude and acceleration interfere with photon return paths, causing transient signal loss and under- or overestimation of HR across all devices. These results align with previous research showing that signal decorrelation and motion-induced baseline drift are the main reasons for PPG errors during vigorous activity [20,21,31]. Interestingly, accuracy improved during graded treadmill exercise, where the cyclic, repeatable gait pattern allowed algorithms to average signals over time and filter periodic noise [40,41]. Therefore, PPG accuracy relies not just on movement intensity but also on kinematic consistency and algorithmic flexibility. This suggests that activity-specific calibration and motion-frequency-aware filtering could enhance wearable HR accuracy in dynamic settings.

### 4.5. Impact of Anatomical Placement on Accuracy: Intra-Device (Whoop 4.0) Comparisons

A notable strength of this study was the intra-device comparison using three identical Whoop 4.0 units worn simultaneously on the wrist, forearm, and upper arm, effectively isolating anatomical placement as the only variable. This method eliminated confounding factors such as hardware, firmware, or arm dominance differences, providing direct evidence that site-specific mechanical and physiological factors mainly influence variations in PPG accuracy. Across all activity levels, the upper-arm placement consistently yielded the lowest error and the highest agreement with the chest-strap reference, followed by the forearm, while the wrist showed the greatest deviations. This pattern of accuracy supports the idea that proximity to the trunk, increased tissue stability, and reduced peripheral acceleration help produce more reliable optical signals [21,31]. The enhanced performance of proximal placements aligns with established optical-physiology principles: thicker tissue layers, greater vascular depth, and higher perfusion stability improve photon scattering and absorption efficiency, while increased muscle mass minimizes relative sensor-skin displacement [13,14,32]. In contrast, wrist regions undergo greater mechanical deformation during flexion and extension and contain more tendinous and bony structures. These differences alter the local refractive index and decrease signal coherence [31]. The intra-device findings further show that firmware or signal-processing adjustments alone cannot fully compensate for anatomical instability; even with identical hardware and algorithms, distal placements still remained significantly less accurate.

These findings have both engineering and clinical implications. From a design perspective, next-generation wearables might incorporate adaptive LED power modulation, accelerometer-synchronized motion feedback, or auto-calibration algorithms to dynamically accommodate site-specific optical conditions [40,41]. Clinically, recommending proximal placement (forearm or upper arm) could improve HR tracking accuracy during rehabilitation, steady-state exercise, or daily activity monitoring, especially when precision is vital for workload assessment or safety monitoring.

## 5. Limitations

This study has several limitations to consider when interpreting the results. All participants were healthy, young, and physically active adults (N = 28), which limits external validity to older adults, individuals with darker skin tones, higher adiposity, arrhythmias, or cardiovascular disease. Since this was a within-subject design where each participant wore all devices at different anatomical sites simultaneously, the results should mainly be seen as relative comparisons between devices and placements, rather than definitive measures of accuracy for all demographic or clinical groups. Future research should involve a more diverse population, including a wider range of skin tones, BMIs, and tissue compositions, to more accurately assess ecological validity [1,4,6,42]. Testing was conducted in a controlled laboratory environment with standardized lighting, temperature, and humidity, which may not reflect the environmental variability experienced during daily life or outdoor training [13,14]. Additionally, activity phases were short, and performance during longer or continuous exercise sessions was not evaluated. Although the study included activities of different intensities, it was limited to walking, cycling, and a plyometric bodyweight task; other exercise modalities, such as swimming, rowing, or contact sports, might pose different motion artifacts and sensor challenges. Variability between devices of the same model was also not examined. Furthermore, all data were transmitted via Bluetooth through a single application, and although connection stability was monitored, transient signal dropouts or latency could not be entirely ruled out.

Device placement was standardized using consistent anatomical landmarks, and all Whoop sensors were positioned on the same arm for methodological consistency. However, the potential effects of arm dominance were not examined. Additionally, factors such as skin tone (melanin content), hair density, skin temperature, and tissue composition—known to affect PPG signal-to-noise ratio [13,14,42]—were not measured, which could confound accuracy estimates [14]. Furthermore, the Polar H10 was chosen as the reference standard due to its established validity. However, a full ECG was not used simultaneously; therefore, any minor deviations of the Polar H10 from accurate HR would be reflected in the wearable comparisons. For the burpee and subsequent recovery phase, HR was analyzed as a single combined interval, which may mask differences in accuracy between active high-intensity effort and post-exercise recovery phases. Future studies should address these limitations by including more diverse populations, longer-duration activities, and direct ECG validation across a broader range of real-world conditions.

## 6. Practical Implications

Placing sensors proximally on the forearm and upper arm yields more stable and accurate PPG signals than wrist sites, due to reduced motion and improved optical coupling between the sensor and the skin [21,31,36]. Compared to traditional ECG, which requires multiple skin electrodes, conductive gel, and lead-wire attachment, PPG wearables provide a comfortable, cable-free alternative suitable for long-term and ambulatory monitoring [10,11]. While ECG remains the gold standard for clinical diagnostics and arrhythmia detection, it is less practical for daily activity tracking or community-based cardiac rehabilitation. In contrast, upper-arm and forearm PPG devices can accurately measure HR during low-to-moderate intensity activities, enabling safer exercise prescriptions and remote supervision in tele-rehabilitation settings [8,9]. For athletic and recreational populations, proximally worn PPG sensors can effectively monitor HR during warm-ups and steady-state training. However, caution is warranted when interpreting HR during ballistic or high-intensity movements, as rapid limb acceleration can cause optical instability and signal dropout. Future wearable designs should include adaptive LED control, multi-wavelength sensing, and accelerometer-based correction to reduce these artifacts and improve usability across different activity intensities [40,41].

## 7. Conclusions

This study demonstrated that PPG accuracy varies with activity intensity and anatomical placement. Heart rate measurements were most accurate at rest and during steady-state activity but decreased during rapid, high-impact movements due to motion artifacts and sensor-skin instability. Proximal placements (upper arm and forearm) consistently outperformed wrist sites because of less motion, more stable tissue, and deeper blood vessels, which can improve optical signal quality. The Polar Verity Sense and Whoop-upper arm had the closest agreement with the Polar H10, confirming that sensor location significantly impacts wearable accuracy. The intra-device comparison (between Whoop devices) also suggested that placement alone can affect performance, regardless of sensor design. These findings support the use of proximal arm positions as practical and reliable alternatives to chest straps for clinical monitoring, cardiac rehab, and steady-state training. However, caution is advised when interpreting wrist-based measurements during active or ballistic exercises, where optical instability remains a limiting factor.

## Figures and Tables

**Table 1:** Participant characteristics (N = 28; 14 males, 14 females). Values are presented as mean ± standard deviation (SD).

Age (years)	23.75 ± 1.11
Height (cm)	171.50 ± 8.00
Weight (kg)	71.74 ± 8.92
Body Mass Index (kg/m^2^)	24.36 ± 2.44
Body Fat Percentage (%)	21.99 ± 6.79

**Table 2:** Mean absolute percentage error (MAPE) of wearable heart rate monitors compared with the Polar H10 chest strap during rest, warm-up, burpees, and the Modified Bruce protocol. Values are presented as mean ± standard deviation (SD).

Device	Rest	Warm-Up	Burpees	Modified Bruce
Verity Sense	1.57 ± 0.77%	3.87 ± 1.92%	16.32 ± 23.65%	1.89 ± 1.21%
Garmin Forerunner	2.11 ± 1.49%	6.88 ± 6.95%	25.48 ± 11.18%	4.29 ± 6.76%
Whoop-upper arm	2.50 ± 0.87%	2.91 ± 2.21%	12.07 ± 24.26%	1.92 ± 1.15%
Whoop-forearm	2.81 ± 0.87%	3.51 ± 1.96%	17.75 ± 24.87%	2.55 ± 3.28%
Whoop-wrist	2.93 ± 1.02%	5.59 ± 4.57%	17.23 ± 21.51%	5.95 ± 5.03%

**Table 3:** Bland–Altman analysis comparing HR data from wearable monitors with the Polar H10 chest strap across activity conditions. Mean bias represents the average difference in HR (bpm), and the 95% LOA indicates the range within which most differences fall. “Proportional bias” refers to whether error magnitude varies with HR intensity, and “Systematic bias” indicates consistent over- or underestimation.

Activity	Device	Mean Bias (bpm)	95% Limits of Agreement (LOA, bpm)	Proportional Bias	Systematic Bias
Rest	Verity Sense	0.29	−0.47 to 1.04	No	No
	Garmin Forerunner	−0.92	−10.10 to 8.27	No	No
	Whoop-upper arm	0.71	−0.28 to 1.70	No	No
	Whoop-forearm	0.77	−0.20 to 1.73	No	No
	Whoop-wrist	0.73	−0.47 to 1.93	No	No
Warm-Up	Verity Sense	0.49	−2.38 to 3.35	No	No
	Garmin Forerunner	4.16	−10.73 to 19.05	No	No
	Whoop-upper arm	0.53	−2.94 to 4.00	No	No
	Whoop-forearm	0.51	−1.77 to 2.79	Yes	Yes
	Whoop-wrist	1.77	−8.23 to 11.77	No	No
Burpees	Verity Sense	6.48	−38.62 to 51.58	Yes	Yes
	Garmin Forerunner	33.16	−10.03 to 76.34	No	No
	Whoop-upper arm	−0.58	−38.53 to 37.36	Yes	Yes
	Whoop-forearm	9.60	−45.12 to 64.32	No	No
	Whoop-wrist	11.39	−30.80 to 53.58	No	No
Modified Bruce Protocol	Verity Sense	0.03	−1.99 to 2.04	No	No
	Garmin Forerunner	2.94	−16.69 to 22.57	No	No
	Whoop-upper arm	0.77	−1.95 to 3.49	Yes	No
	Whoop-forearm	1.12	−2.56 to 4.80	Yes	Yes
	Whoop-wrist	2.90	−8.59 to 14.38	Yes	Yes

**Table 4:** Concordance correlation coefficients (ρc) for wearable heart rate monitors compared with the Polar H10 chest strap during rest, warm-up, burpees, and the Modified Bruce protocol. Higher values indicate stronger agreement between devices.

Device	Rest	Warm-Up	Burpees	Modified Bruce
Verity Sense	0.9990	0.9912	0.1343	0.9968
Garmin Forerunner	0.9961	0.7677	0.1521	0.7323
Whoop-upper arm	0.9964	0.9879	0.4605	0.9922
Whoop-forearm	0.9960	0.9940	0.1021	0.1021
Whoop-wrist	0.9956	0.8910	0.3100	0.8514

**Table 5:** Deming regression analysis comparing HR from wearable devices with the Polar H10 chest strap across all activity conditions. Slopes near 1.0 indicate proportional agreement, and intercepts near 0 indicate minimal systematic bias. 95% confidence intervals (CI) are reported for slope and intercept.

Activity	Device	Slope (95% CI)	Intercept (95% CI)	Proportional Bias	Systematic Bias
Rest	Verity Sense	1.0026 (0.988–1.017)	−0.46 (−1.45–0.52)	No	No
	Garmin Forerunner	0.9940 (0.960–1.028)	0.46 (−1.93–2.85)	No	No
	Whoop-upper arm	0.9999 (0.981–1.019)	−0.71 (−2.04–0.62)	No	No
	Whoop-forearm	0.9957 (0.977–1.014)	−0.47 (−1.78–0.83)	No	No
	Whoop-wrist	0.9881 (0.965–1.011)	0.09 (−1.50–1.68)	No	No
Warm-Up	Verity Sense	0.9542 (0.911–0.997)	3.73 (−0.25–7.71)	Yes	No
	Garmin Forerunner	1.1054 (0.834–1.377)	−13.86 (−31.9–4.1)	No	No
	Whoop-upper arm	1.0008 (0.943–1.059)	−0.60 (−6.0–4.8)	No	No
	Whoop-forearm	0.9507 (0.919–0.982)	4.03 (1.10–6.96)	Yes	Yes
	Whoop-wrist	0.9311 (0.769–1.093)	4.57 (−10.3–19.4)	No	No
Burpees	Verity Sense	0.1230 (−0.08–0.33)	116.19 (89.0–143.4)	No	Yes
	Garmin Forerunner	0.5303 (0.23–0.83)	32.55 (−9.9–75.0)	Yes	No
	Whoop-upper arm	0.4546 (0.23–0.68)	76.87 (44.5–109.3)	Yes	Yes
	Whoop-forearm	0.4259 (0.04–0.81)	70.71 (14.7–126.7)	Yes	Yes
	Whoop-wrist	0.4446 (0.17–0.72)	66.30 (28.3–104.3)	Yes	Yes
Modified Bruce Protocol	Verity Sense	0.9787 (0.950–1.008)	2.62 (−1.0–6.2)	No	No
	Garmin Forerunner	0.7936 (0.65–0.94)	22.73 (4.8–40.7)	Yes	Yes
	Whoop-upper arm	0.9568 (0.92–0.99)	4.60 (−0.0–9.2)	Yes	No
	Whoop-forearm	0.9358 (0.89–0.98)	6.86 (0.9–12.8)	Yes	Yes
	Whoop-wrist	0.7936 (0.65–0.94)	22.73 (4.8–40.7)	Yes	Yes

## Data Availability

The data presented in this study are available on request from the corresponding author. The data are not publicly available due to privacy or ethical restrictions.
